# Novel *KAT6B-KANSL1* Fusion Gene Identified by RNA Sequencing in Retroperitoneal Leiomyoma with t(10;17)(q22;q21)

**DOI:** 10.1371/journal.pone.0117010

**Published:** 2015-01-26

**Authors:** Ioannis Panagopoulos, Ludmila Gorunova, Bodil Bjerkehagen, Sverre Heim

**Affiliations:** 1 Section for Cancer Cytogenetics, Institute for Cancer Genetics and Informatics, The Norwegian Radium Hospital, Oslo University Hospital, Oslo, Norway; 2 Centre for Cancer Biomedicine, Faculty of Medicine, University of Oslo, Oslo, Norway; 3 Department of Pathology, The Norwegian Radium Hospital, Oslo University Hospital, Oslo, Norway; 4 Faculty of Medicine, University of Oslo, Oslo, Norway; Ospedale Pediatrico Bambino Gesu’, ITALY

## Abstract

Retroperitoneal leiomyoma is a rare type of benign smooth muscle tumor almost exclusively found in women and with histopathological features similar to uterine leiomyomas. The pathogenesis of retroperitoneal leiomyoma is unclear and next to nothing is known about the cytogenetics and molecular genetics of the tumor. Here we present the first cytogenetically analyzed retroperitoneal leiomyoma. It had a t(10;17)(q22;q21) as the sole chromosomal abnormality. Using RNA-Sequencing and the ‘grep’ command to search the fastq files of the sequence data we found that the translocation resulted in fusion of the genes *KAT6B* (10q22) with *KANSL1* (17q21). RT-PCR together with direct (Sanger) sequencing verified the presence of a *KAT6B-KANSL1* fusion transcript. No reciprocal *KANSL1-KAT6B* transcript was amplified suggesting that it was either absent or unexpressed. The *KAT6B-KANSL1* fusion transcript consists of exons 1 to 3 of *KAT6B* and exons 11 to 15 of *KANSL1*, is 3667 bp long, has a 1398 bp long open reading frame, and codes for a 466 amino acid residue protein. The corresponding KAT6B-KANSL1 protein contains the NEMM domain (including the linker histone H1/H5, domain H15) of KAT6B and the PEHE domain of KANSL1. The function of the fusion protein might be regulation of transcription with an affinity for chromatin (linker histone H1/H5) and interaction with the HAT domain of KAT8 (PEHE domain). The tumor expressed *HMGA2* and *HMGA1* even though 12q14-15 and 6p looked normal by G-banding analysis. The tumor also expressed *MED12* in the absence of exon 2 mutations. Overall, the data show that the examined retroperitoneal leiomyoma resembles a subset of uterine leiomyomas in terms of histology and genetics.

## Introduction

Leiomyomas are benign tumors that display smooth muscle differentiation, the most common type being uterine leiomyoma. Extrauterine leiomyomas are rare, may arise in any anatomic site, and may be difficult to distinguish from malignancies [1]. Unusual growth patterns have been reported; they include benign metastasizing leiomyoma, disseminated peritoneal leiomyomatosis, intravenous leiomyomatosis, parasitic leiomyoma, and retroperitoneal leiomyoma [1].

Retroperitoneal leiomyomas are almost exclusively found in women [2–4]. They may enlarge considerably before they become symptomatic and are often detected incidentally at a routine check-up or during autopsy [5]. Common symptoms include discomfort, fatigue, and back pain [4]. The pathogenesis is unknown and it is not clear whether the tumors represent metastatic or primary lesions [6] and whether they arise from hormonally sensitive smooth muscle elements [2] or from the embryonal remnants of Müllerian or Wolffian ducts [7]. In terms of histology, retroperitoneal leiomyomas resemble their uterine counterparts in often having hyaline fibrosis, alternating myxoid change or trabecular matterns, and positivity for estrogen and progesterone receptors [2–4]. They show low mitotic activity with little to no atypia, no necrosis, and immunohistochemical features consistent with smooth muscle tumors with positive staining for desmin and smooth muscle–specific actin (SMA) but negative staining for C-KIT and CD34; the latter finding rules out gastrointestinal stromal tumor [4].

No information about the cytogenetics and molecular genetics of retroperitoneal leiomyomas is given in the “Mitelman database of chromosome aberrations in cancer” (http://cgap.nci.nih.gov/Chromosomes/Mitelman, database last updated on May 13, 2014) nor is any provided in the 2013 edition of “WHO classification of tumours of soft tissue and bone” [8].

Recently, however, mutations in exon 2 of the mediator complex subunit 12 (*MED12*) gene were found in 10 out of 29 (34%) leiomyomas/leiomyomatoses in pelvic/retroperitoneal sites [9]. Since mutations in exon 2 of *MED12* are found also in the majority (50–80%) of uterine leiomyomas [9–13], Schwetye et al. [9] concluded that “smooth muscle tumors in pelvic/retroperitoneal sites are subject to the same mutational changes as those of uterine myometrium, and [that] these mutations may precede the gross or histological development of a leiomyoma” [9].

Most uterine leiomyomas are cytogenetically characterized by the presence of one or more of the following cytogenetic aberrations: t(12;14)(q15;q23–24), del(7)(q21.2q31.2), rearrangements involving 6p21, 10q, and 1p, trisomy 12, deletions of 3q, and changes of the X chromosome [14]. Besides, 34 out of 495 (7%) uterine leiomyomas have aberrations involving the q arm of chromosome 10 and in 25 of these tumors the breakpoint targets chromosome band 10q22 (http://cgap.nci.nih.gov/Chromosomes/Mitelman, Database last updated on May 13, 2014). Moore et al. [15] studied four uterine leiomyomas with rearrangements of 10q and 17q and found disruption of the *KAT6B* gene in 10q22 (also known as *MORF* and *MYST4*) in all of them. They mapped the breakpoint in 17q21 in three tumors but detected no fusion gene. The *KAT6B* gene encodes a member of the MYST family of histone acetylases (histone acetyltrasferase) and was shown to be in-frame fused to *CREBBP* in acute myeloid leukemia with t(10;16)(q22;p13) [16].

Here we present the first cytogenetically analyzed retroperitoneal leiomyoma. The tumor had a t(10;17)(q22;q21) as the sole karyotypic aberration. Using RNA-sequencing (RNA-Seq) and the “grep” command we could demonstrate that the molecular consequence of the translocation was fusion of *KAT6B* with the *KANSL1* gene (official full name: KAT8 regulatory NSL complex subunit 1) from 17q21. The tumor was also investigated for expression of *HMGA2*, *HMGA1*, and *MED12* as well as for possible mutations in exon 2 of *MED12*.

## Materials and Methods

### Ethics Statement

The study was approved by the regional ethics committee (Regional komité for medisinsk forskningsetikk Sør-Øst, Norge, http://helseforskning.etikkom.no), and written informed consent was obtained from the patient.

### Case History

**Figure 1:**
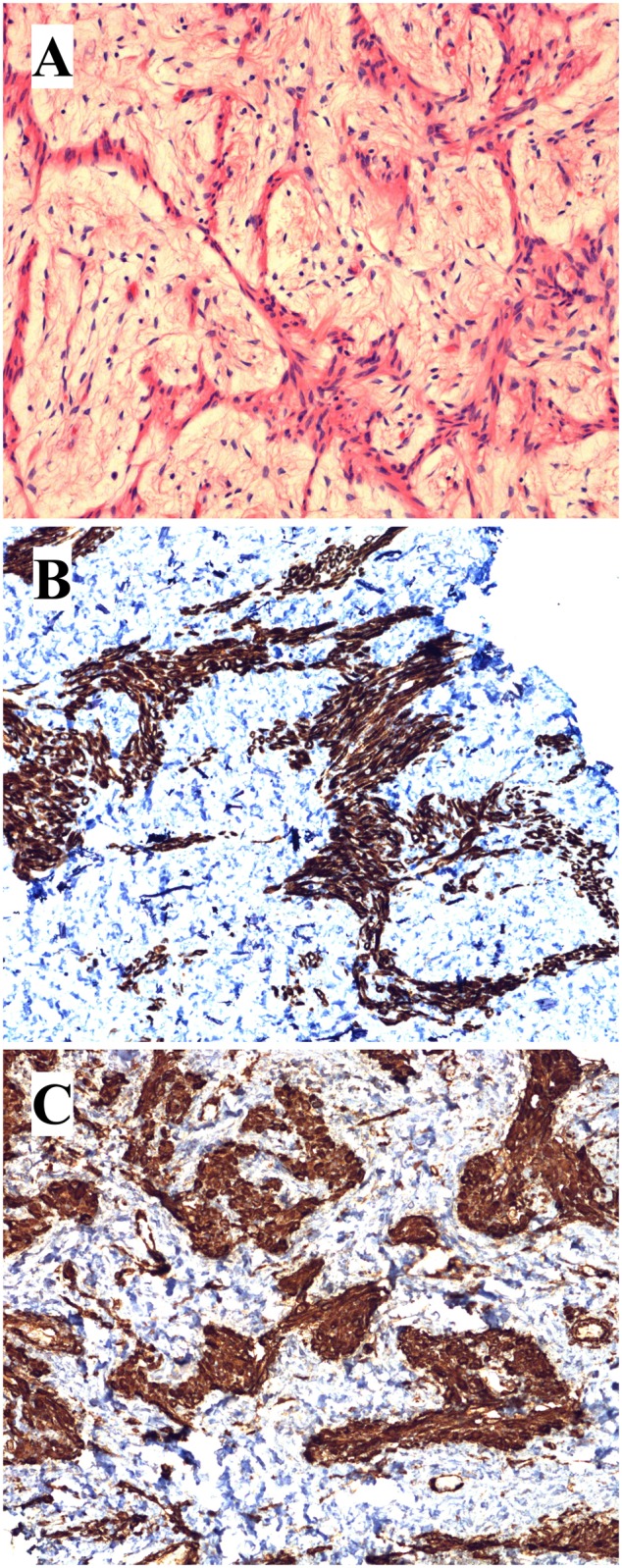
Histological examination of the retroperitoneal leiomyoma. A) H&E-stained slide showing the tumor spindle cells with eosinophilic cytoplasm without atypia surrounded by loose fibrous stroma. B) Immunoexpression of desmin. C) Immunoexpression of SMA.

A 45-year-old previously healthy woman presented with abdominal discomfort and a large retroperitoneal tumor was detected and resected. Microscopic examination showed fascicles of long spindle cells with eosinophilic cytoplasm surrounded by a loose fibrous matrix (Fig. 1). Immunohistochemistry demonstrated positive staining for SMA, desmin, and estrogen and progesterone receptors, but was negative for the cytokeratin cocktail AE1/AE3, CD68, CD99, CD117, EMA, HMB-45, S-100, and Melan-A. Neither atypia nor necrosis was found. There were very few mitotic figures (0–1/10 high power fields). The histological diagnosis was leiomyoma. After surgery the patient has had no recurrence (4 years).

### G-banding and Karyotyping

Fresh tumor tissue was received and analyzed cytogenetically as part of our diagnostic routine. The sample was disaggregated mechanically and enzymatically with collagenase II (Worthington, Freehold, NJ, USA). The resulting cells were cultured and harvested using standard techniques. Chromosome preparations were G-banded with Wright stain and examined. The karyotype was written according to The International System for Human Cytogenetic Nomenclature (ISCN) 2009 guidelines [17].

### High-throughput Paired-end RNA-sequencing

**Figure 2:**
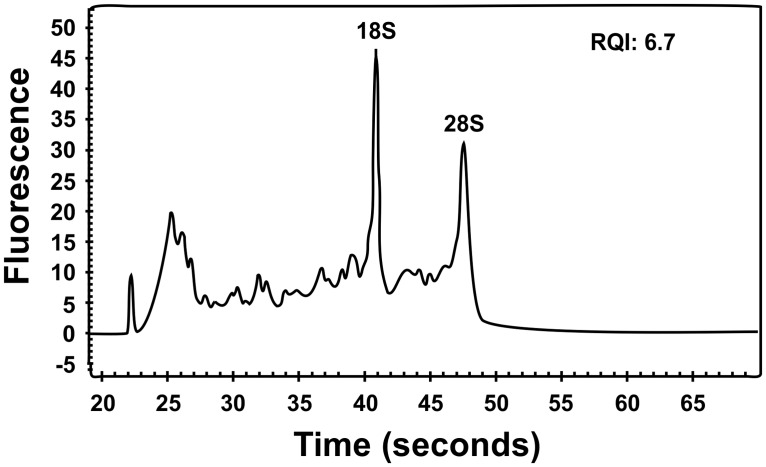
Electropherogram showing the quality of the extracted RNA from the retroperitoneal leiomyoma which was used for RNA-Seq and RT-PCR experiments. The RNA was run on the Experion automated electrophoresis system using an Experion RNA StdSens analysis chip. The RQI value 6.7 was automatically calculated by the Experion.

Tumor tissue adjacent to that used for cytogenetic analysis and histologic examination had been frozen and stored at -80°C. Total RNA was extracted using Trizol reagent according to the manufacturer’s instructions (Invitrogen, Oslo, Norway) with a homogenizer (Omni THQ Digital Tissue Homogenizer, Kennesaw, GA, USA). The RNA quality was evaluated using the Experion Automated Electrophoresis System (Bio-Rad Laboratories, Oslo, Norway). The RNA Quality Indicator (RQI) was 6.7 and the electropherogram of the sample obtained by the Experion system is shown in Fig. 2. Three µg of total RNA were sent for high-throughput paired-end RNA-sequencing at the Norwegian Sequencing Centre, Ullevål Hospital (http://www.sequencing.uio.no/). The RNA was sequenced using an Illumina HiSeq 2000 instrument and the Illumina software pipeline was used to process image data into raw sequencing data. The regular TruSeq library preparation protocol was used (http://support.illumina.com/downloads/truseq_rna_sample_preparation_guide_15008136.ilmn) and the reads obtained had a length of 100 base pairs. A total of 74 million reads were obtained. The quality of the raw sequence data was assessed using FastQC software (http://www.bioinformatics.babraham.ac.uk/projects/fastqc/). The softwares FusionMap (http://www.omicsoft.com/fusionmap/) [18] and FusionFinder (http://bioinformatics.childhealthresearch.org.au/software/fusionfinder/) [19] were used for the discovery of fusion transcripts. In addition, the “grep” command (http://en.wikipedia.org/wiki/Grep) was used to search the fastq files of the sequence data (http://en.wikipedia.org/wiki/FASTQ_format) for *KAT6B* sequence (NM_012330 version 3). FusionMap was run on a PC with Windows 7 Professional as the operative system. FusionFinder and “grep” command were run on a PC with Bio-Linux 7 as the operating system [20].

### Molecular Genetic Analyses

Two µg of total RNA were reverse-transcribed in a 20 µL reaction volume using iScript Advanced cDNA Synthesis Kit for RT-qPCR according to the manufacturer’s instructions (Bio-Rad Laboratories). The cDNA was diluted to 50 µL and 1 µL was used as template in subsequent PCR assays. The 25 µL PCR volumes contained 12.5 µL of Premix Taq (Takara Bio Europe/SAS, Saint-Germain-en-Laye, France), 1 µL of diluted cDNA, and 0.2 µM of each of the forward and reverse primers. The PCRs were run on a C-1000 Thermal cycler (Bio-Rad Laboratories). The PCR conditions for all amplifications except *HMGA1* were: an initial denaturation at 94°C for 30 sec followed by 35 cycles of 7 sec at 98°C and 2 min at 68°C, and a final extension for 5 min at 68°C. For the detection of the *KAT6B-KANSL1* fusion transcript, two forward *KAT6B* primers, MORF-1234F (5′-GCT TAG ATG GCA AAG GGG CAC CTC-3′) and MORF-1261F (5′-ATC CCA GTG CAT TCC CAT CCT CG-3′), and two reverse *KANSL1* primers, KANSL1–2969R1 (5′-GTG GCA CAC TCG TGG TCC ACA GC-3′) and KANSL1–2900R1 (5′-AGG CTG CGT CGG ATA GGT CCT CA-3′), were used. The following primer combinations were applied: 1) MORF-1234F together with the primer KANSL1–2969R1, 2) MORF-1234F together with the primer KANSL1–2900R1, 3) MORF-1261F together with the primer KANSL1–2969R1, and 4) MORF-1261F together with the primer KANSL1–2900R1.

For the detection of a possible reciprocal *KANSL1-KAT6B*, nested PCR was used. In the first round, the *KANSL1* forward primer KANSL1–2341F1 (5′-GGC ACA AAT TGG TCA GCT CCT TCC T-3′) and the reverse *KAT6B* primer MORF-1591R1 (5′-GAA AGT GGT GGG TCA CAG CAT TCC A-3′) were used. One microliter of the first PCR was used as template in the nested PCR together with primers KANSL1–2407F1 (5′-GGA CCC ACA GGC AGC ACT TAG ACG-3′) and MORF-1483R1 (5′-CAT TCG ATG CAC TGC CAC CTT AAG G-3′).

For the expression of *HMGA2*, two PCR amplifications were performed. The primer set HMGA2–846F1 (5′-CCA CTT CAG CCC AGG GAC AAC CT-3′)/HMGA2–1021R (5′-CCT CTT GGC CGT TTT TCT CCA GTG-3′) was used for the amplification of transcripts of *HMGA2* exons 1–3. The primer set HMGA2–846F1/HMGA2–1112R (5′-CCT CTT CGG CAG ACT CTT GTG AGG A-3′) was used for transcripts of *HMGA2* containing exons 1–5. For the expression of *HMGA1*, the primers HMGA1–284F1 (5′-CAG CCA TCA CTC TTC CAC CTG C-3′) and HMGA1–648R1 (5′-CTG TCC AGT CCC AGA AGG AAG CT-3′) were used. The PCR conditions were: an initial denaturation at 94°C for 30 sec followed by 35 cycles of 7 sec at 98°C, 30 sec at 55°C and 1 min at 72°C, and a final extension for 5 min at 72°C.

For the expression of *MED12* and the detection of possible mutation in exon 2 of *MED12*, the primers MED12-Ex1-F (5′-TTA CCC TCA GGA CCC CAA ACA G-3′) and MED12-Ex3-R (5′-TGC AAT AAT GCT GCT GAA GTT GG-3′) were used.

Three µL of the PCR products were stained with GelRed (Biotium), analyzed by electrophoresis through 1.0% agarose gel, and photographed. The remaining PCR products were purified using the Qiagen PCR purification kit (Qiagen) and direct sequencing was performed using the light run sequencing service of GATC Biotech (http://www.gatc-biotech.com/en/sanger-services/lightrun-sequencing.html). The BLAST software (http://www.ncbi.nlm.nih.gov/BLAST/) was used for computer analysis of sequence data.

### Immunohistochemistry

To detect the HMGA2 protein, immunostaining was performed as described previously [21].

## Results

### G-banding

**Figure 3:**
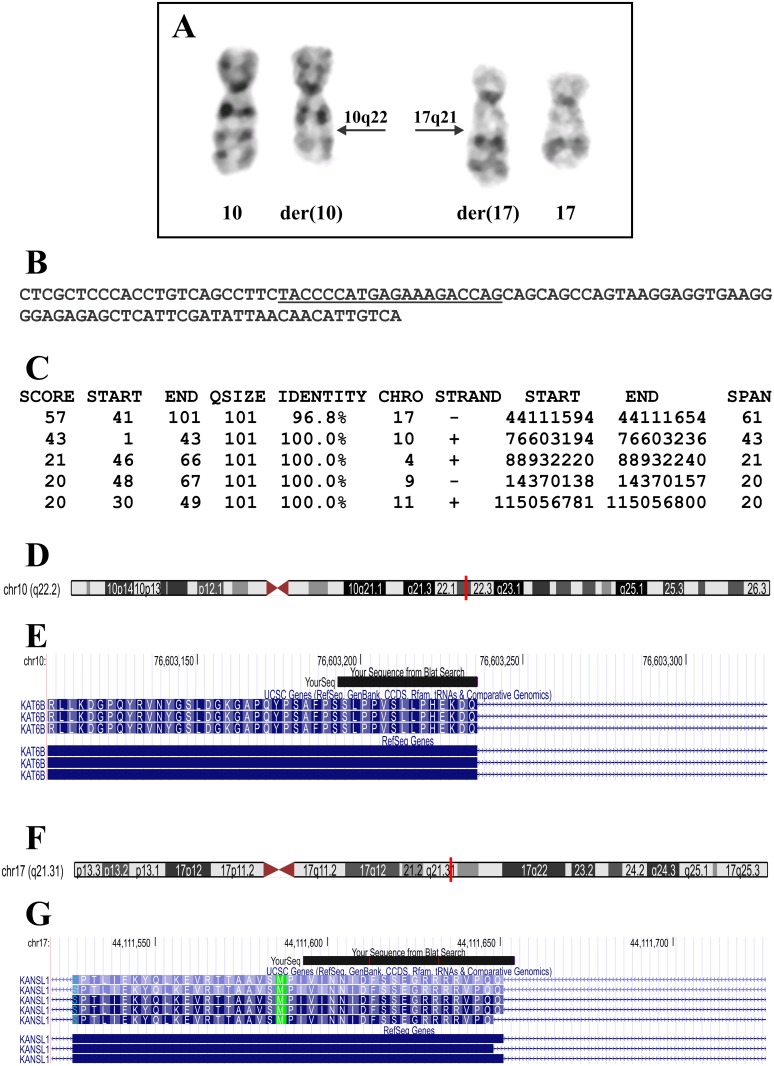
Cytogenetic results and bioinformatic analyses of the RNA-Seq data of the retroperitoneal leiomyoma. A) Partial karyotype showing the der(10)t(10;17)(q22;q21) and der(17)t(10;17)(q22;q21) together with the corresponding normal chromosome homologs; breakpoint positions are indicated by arrows. B) The 101 bp sequence obtained from the raw data of RNA-Seq using the command “grep”. The search term “TACCCCATGAGAAAGACCAG” is underlined. C) The results of BLAT search genome using the 101 bp sequence (see B) on the human genome browser-hg19 assembly (http://genome-euro.ucsc.edu/cgi-bin/hgGateway). D) Ideogram of chromosome 10 showing that nucleotides 1–43 of the 101 bp sequence (see B) are mapped on band q22.2 (red vertical line). E) The result of BLAT search (your sequence from BLAT search) showed that nucleotides 1–43 are part of the *KAT6B* coding region. F) Ideogram of chromosome 17 showing that nucleotides 41–101 of the sequence from B are mapped on band q21.3 (red vertical line). G) The result of BLAT search (your sequence from BLAT search) shows that the nucleotides are part of the *KANSL1* coding region.

The G-banding analysis yielded a karyotype with a single chromosome abnormality: 46,XX,t(10;17)(q22;q21)[10]/46,XX[5] (Fig. 3A).

### High-throughput Paired-end RNA-sequencing Analysis

FusionMap and FusionFinder identified 609 and 85 potential fusion transcripts, respectively (S1 and S2 Tables), but none of them was related to the chromosome aberration, t(10;17)(q22;q21). Nor was *KAT6B* found to be a partner in any of the detected fusion transcripts (S1 and S2 Tables). *KAT6B* was reported to be targeted in four uterine leiomyomas with a chromosome rearrangement involving 10q22 and 17q21–24 and the breakpoint was mapped to the third intron of the *KAT6B* gene [15]. Since the raw sequence data were in the text-based fastq format, we used the “grep” command-line utility to search for sequences which contained part of the third exon of *KAT6B* (nucleotides 444–1322 in the sequence with accession number NM_012330 version 3). The search term was “TACCCCATGAGAAAGACCAG” that corresponded to the last 20 nucleotides of exon 3 of *KAT6B* (nt 1303–1322 in the sequence with accession number NM_012330 version 3). The search term extracted only the following 101 bp long sequence: “CTCGCTCCCACCTGTCAGCCTTCTACCCCATGAGAAAGACCAGCAGCAGCCAGTAAGGAGGTGAAGGGGAGAGAGCTCATTCGATATTAACAACATTGTCA” (Fig. 3B). BLAT of this sequence on the human genome browser-hg19 assembly (http://genome-euro.ucsc.edu/cgi-bin/hgGateway) showed that nucleotides 1–43 mapped on chromosome 10 at position 76603194–76603236, on 10q22.2 in a coding region of *KAT6B*, whereas nucleotides 41–101 mapped on chromosome 17 at position 44111594–44111654, on 17q21.31 in coding region of *KANSL1* (Fig. 3C‐G). These data were verified when we used the BLAST algorithm (http://blast.ncbi.nlm.nih.gov/Blast.cgi) to compare the 101 bp sequence with the *KAT6B* reference sequence NM_012330 version 3 and the *KANSL1* reference sequence NM_015443 version 3. The nucleotides “CTCGCTCCCACCTGTCAGCCTTCTACCCCATGAGAAAGACCAG” correspond to nt 1280–1322 of the *KAT6B* reference sequence NM_012330 version 3 and the nucleotides “CAGCAGCCAGTAAGGAGGTGAAGGGGAGAGAGCTCATTCGATATTAACAACATTGTCA” correspond to nt 2715–2772 of the *KANSL1* reference sequence NM_015443 version 3.

Thus, the 101 bp sequence which was obtained with the “grep”command was part of a chimeric *KAT6B-KANSL1* transcript which could have been generated by the chromosome abnormality t(10;17)(q22;q21) since *KAT6B* is located on 10q22.2 and *KANSL1* is located in 17q21.31.

### Molecular Genetic Confirmation of the KAT6B-KANSL1 Fusion

**Figure 4:**
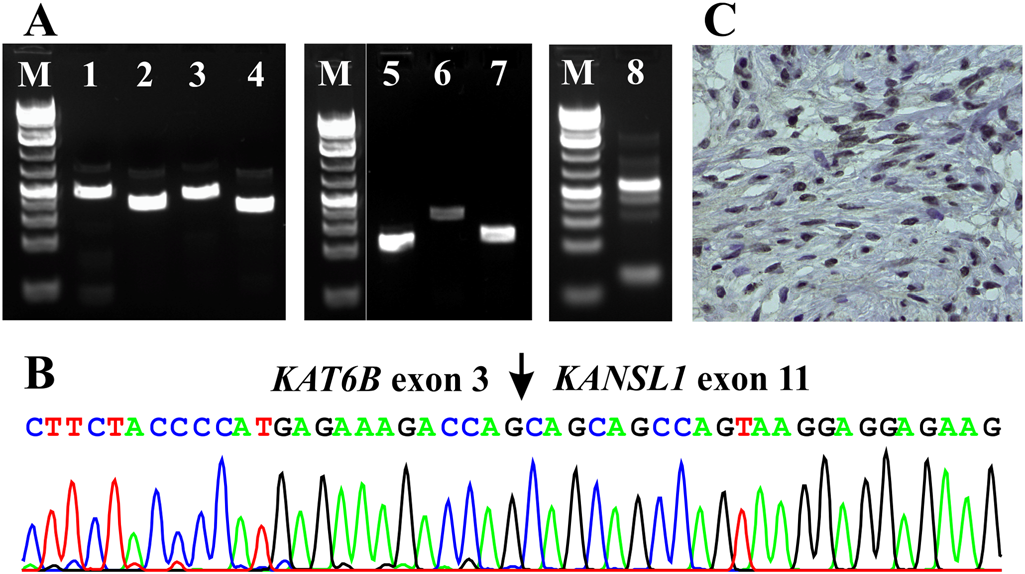
Expression of *KAT6B-KANSL1, HMGA2, HMGA1*, and *MED12* in the retroperitoneal leiomyoma. A) Gel electrophoresis of the amplified products. Lanes 1–4: RT-PCR with primer combinations MORF-1234F/KANSL1–2969R1, MORF1234F/KANSL1–2900R1, MORF-1261F/KANSL1–2969R1, and MORF-1261F/KANSL1–2900R1, respectively. The amplified cDNA fragments indicated the presence of a *KAT6B-KANSL1* fusion transcript in the examined tumor. Lanes 5 and 6: RT-PCR with primer combinations HMGA2–846F1/HMGA2–1021R and HMGA2–846F1/HMGA2–1112R, respectively, showed expression of *HMGA2*. Lane 7: RT-PCR with primers MED12-Ex1-F/MED12-Ex3-R indicated expression of *MED12*. Lane 8: RT-PCR with primer set HMGA1–284F1/HMGA1–648R indicated expression of *HMGA1*. B) Partial sequence chromatogram of the amplified cDNA fragment showing that exon 3 of *KAT6B* is fused to exon 11 of *KANSL1*. C) Tumor tissue showing widespread immunohistochemical nuclear staining for the HMGA2 protein.

To verify the data obtained with the “grep” command, PCR amplifications were performed using two forward *KAT6B* and two reverse *KANSL1* primers corresponding to sequences located upstream and downstream of the putative breakpoint, respectively. RT-PCR with all four primer combinations, MORF-1234F/KANSL1–2969R1, MORF-1234F/ KANSL1–2900R1, MORF-1261F/KANSL1–2969R1, and MORF-1261F/KANSL1–2900R1, amplified cDNA fragments strongly suggesting the presence of a *KAT6B-KANSL1* fusion transcript in the examined tumor (Fig. 4A, lanes 1–4). Sequencing of the amplified cDNA fragment obtained with the MORF-1234F/KANSL1–2969R1 primer set showed that it was a *KAT6B-KANSL1* chimeric cDNA fragment in which exon 3 of *KAT6B* (nucleotide 1322 accession number NM_012330 version 3) was in-frame fused to exon 11 of *KANSL1* (nucleotide 2715 accession number NM_015443 version 3) (Fig. 4B). A reciprocal *KANSL1-KAT6B* transcript was not amplified suggesting that it was absent or not expressed (data not shown).

### Expression of HMGA2 and HMGA1

RT-PCR with the primer sets HMGA2–846F1/HMGA2–1021R and HMGA2–846F1/HMGA2–1112R amplified cDNA fragments from the tumor (Fig. 4A, lanes 5–6). The results indicated that a full length *HMGA2* transcript was expressed in the examined leiomyoma although no chromosome 12q rearrangements were seen cytogenetically. Similarly, RT-PCR with the primer set HMGA1–284F1/HMGA1–648R1 amplified a *HMGA1* cDNA fragment indicating that the *HMGA1* gene was expressed in the tumor (Fig. 4A, lane 8). The immunohistochemical analysis demonstrated nuclear expression of the HMGA2 protein (Fig. 4C).

### Expression of MED12 and Mutations in Exon 2 of MED12

RT-PCR with the primers MED12-Ex1-F/MED12-Ex3-R amplified a 163 bp cDNA fragment which contained part of exon 1, the entire exon 2, and part of exon 3 of the *MED12* gene suggesting that *MED12* is expressed (Fig. 4A, lane 7). Sequencing of the PCR product did not show any mutation in the amplified cDNA fragment of *MED12* (data not shown).

## Discussion

We used RNA sequencing and the “grep” command on raw RNA sequencing data to identify a *KAT6B-KANSL1* fusion gene in a retroperitoneal leiomyoma carrying a t(10;17)(q22;q21) chromosome translocation. The principle of this approach is based on the fact that RNA sequencing data produced on high throughput sequence platforms come as fastq format files (filename.fastq). Fastq file is a text-based format (http://en.wikipedia.org/wiki/FASTQ_format) and can be searched using the “grep” command-line utility (http://en.wikipedia.org/wiki/Grep) to look for specific expressions. By default “grep” displays the lines where matches occur. Using as “specific expression” a sequence of, for example, 20 nucleotides, the “grep” command will display all the lines (sequence reads) which contain the 20 nucleotides of the specific expression. The sequences obtained by “grep” could be further analyzed using BLAST (http://blast.ncbi.nlm.nih.gov/Blast.cgi) and BLAT (http://genome-euro.ucsc.edu/cgi-bin/hgBlat?command=start) algorithms and aligned against a known sequence database such as the human genomic plus transcript database. This approach was used before to detect the fusion transcripts *KAT6A-CREBBP* and *CIC-DUX4* as well as a novel alternative transcript of *CSF1* in an acute myeloid leukemia, a small round cell sarcoma, and tenosynovial giant cell tumors, respectively [22–24].

Our hypothesis was that the translocation of the examined leiomyoma had created a genomic breakpoint in intron 3 of *KAT6B*, similar to what was found in the four uterine leiomyomas reported by Moore et al. [15], one of which had a balanced t(10;17)(q22;q23) as the sole chromosomal abnormality. This would enable a fusion of the first 3 exons of *KAT6B* with a gene located in 17q21 to generate a chimeric transcript. Using the search term “TACCCCATGAGAAAGACCAG” which corresponds to the last 20 nucleotides of exon 3 of *KAT6B* (nt 1303–1322 in the sequence with accession number NM_012330 version 3), the “grep” command displayed a 101 bp chimeric *KAT6B-KANSL1* sequence (Fig. 2B). Subsequent RT-PCR and Sanger sequencing of the PCR amplified fragments verified the *KAT6B-KANSL1* fusion transcript (Fig. 3A-B), whereas the reciprocal *KANSL1-KAT6B* fusion transcript could not be found (data not shown).

The *KAT6B* gene (in the original article the gene name was *MORF*) was identified as a *KAT6A* (also named *MOZ*)-related gene which displays 60% identity and 66% similarity to *MOZ* [25]. The KAT6B protein contains an N-terminal region referred to as the NEMM domain (N‐terminal region in Enok, MOZ or MORF), C4HC3 PHD zinc fingers, a histone acetyltransferase (HAT) domain, an acidic region, and a C-terminal ser/met-rich domain, as does KAT6A [25, 26]. The C-terminal part of the NEMM domain displays sequence similarity to the globular domains of linker histones H1 and H5. These H1‐ and H5‐like regions, known as H15 domains, may mediate self‐association and interaction with core histones and nucleosomes since the globular domains of histones H1 and H5 are known to have similar activities [27, 28]. Linker histone H1 is an essential component of chromatin structure. H1 links nucleosomes into higher order structures. Histone H5 performs the same function as histone H1 and replaces H1 in certain cells (http://www.ebi.ac.uk/interpro/entry/IPR005818).

*KAT6B* was previously shown to be rearranged and fused to *CREBBP* in an acute myeloid leukemia carrying a t(10;16)(q22;p13) chromosome translocation and then both *KAT6B-CREBBP* and *CREBBP-KAT6B* chimeras were expressed [16]. The KAT6B-CREBBP chimeric protein retains the part of *KAT6B* that encodes the N-terminal region NEMM domain, the PHD zinc fingers, two nuclear localization signals, the HAT domain, and a portion of the acidic domain and the CBP protein downstream of codon 29. In the reciprocal *CREBBP-KAT6B* fusion transcript, part of the acidic domain of *MORF*, the ser-rich region, and the highly met-enriched C-terminal part are likely to be driven by the *CREBBP* promoter [16]. Recently, constitutional mutations in *KAT6B* were found to cause Genitopatellar syndrome (GPS) and the Say-Barber-Biesecker variant of Ohdo syndrome (SBBYSS) [29, 30]. Mutations leading to GPS occur in the proximal portion of the last exon and lead to the expression of a protein without a C-terminal domain. Mutations leading to SBBYSS occur either throughout the gene, leading to nonsense-mediated decay, or more distally in the last exon [31]. *KAT6B* was also found to be disrupted in a boy with a Noonan syndrome–like phenotype carrying a balanced constitutional de novo chromosome translocation, t(10;13)(q22.3;q34) [32]. The breakpoint in 10q22.3 disrupted the *KAT6B* gene within intron 3 after the first coding exon, i.e., the same intron in which the breakpoint was mapped in four uterine leiomyomas with rearrangement of the *KAT6B* gene [15]. A 50% reduction of mRNA expression levels of *KAT6B* was confirmed by quantitative RT-PCR in the patient’s leukocytes [32]. Haploinsufficiency of *KAT6B* impaired histone acetylation and was the underlying cause of the phenotype. The MAPK signaling pathway represents a major target of *KAT6B* activity and *KAT6B* insufficiency causes enhanced phosphorylation of several genes in this pathway [32].

The *KANSL1* gene encodes, according to the gene database, a nuclear protein that is a subunit of two protein complexes, MLL1 and NSL1, involved in histone acetylation. The corresponding protein in Drosophila interacts with K(lysine) acetyltransferase 8, which is also a subunit of both the MLL1 and NSL1 complexes (http://www.ncbi.nlm.nih.gov/gene/284058). The KANSL1 protein consists of 1105 amino acid residues and contains at its C-terminal end the PEHE domain which interacts with the HAT domain of KAT8 (also known as MOF and MYST1) [33, 34]. Recently, a study on the nonspecific lethal (NSL) complex showed that, within it, KANSL1 acts as a scaffold protein interacting with four other subunits, including WDR5, which in turn binds KANSL2 [35]. Mutations in *KANSL1* cause the Koolen de Vries syndrome (also known as the chromosome 17q21.31 microdeletion syndrome) (http://www.17q21.com/en/index.php).

**Figure 5:**
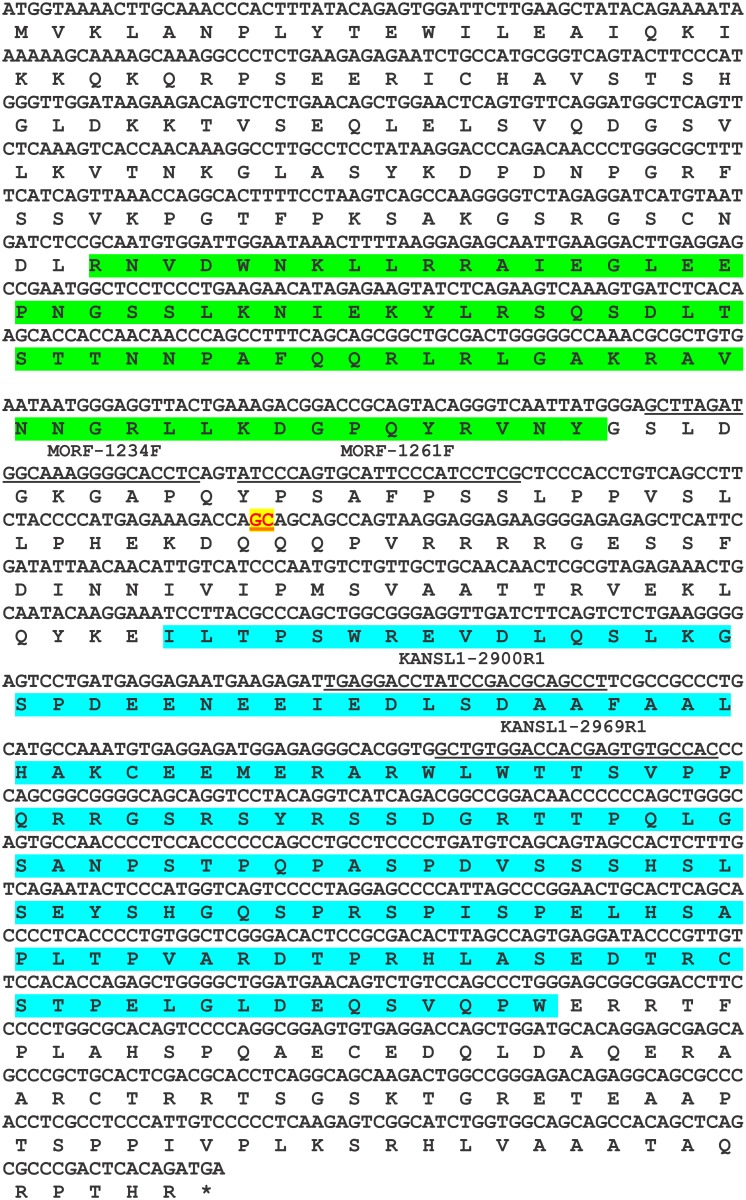
The putative 1398 bp open reading frame of *KAT6B-KANSL1* and the coded 466 amino acid residue protein. The junction GC is in red with yellow background and underlined. The four primers MORF-1234F, MORF-1261F, KANSL1–2900R1, and KANSL1–2969R1 are underlined. The H15 linker histone H1/H5 globular (H15) domain profile (green) and PEHE domain (blue) are shown.

In the present study, the *KAT6B-KANSL1* fusion transcript, composed of exons 1 to 3 of *KAT6B* and exons 11 to 15 of *KANSL1*, is 3667 bp long, has a 1398 bp long open reading frame, and codes for a 466 amino acid residue protein (Fig. 5). The putative KAT6B-KANSL1 fusion protein contains the NEMM domain (including the linker histone H1/H5, domain H15) of KAT6B and the PEHE domain of KANSL1 suggesting an affinity for chromatin (linker histone H1/H5) and interaction with the HAT domain of KAT8 (PEHE domain). The overall function might be regulation of transcription.

At the genomic level, *KAT6B* is transcribed from centromere to telomere whereas the transcription of *KANSL1* proceeds in the opposite direction from telomere to centromere. Hence, the formation of *KAT6B-KANSL1* fusion is only possible if another genomic aberration, possibly an inversion, occurs in addition to the chromosome translocation. Such an event would be analogous to the formation of *EWSR1-DDIT3* in myxoid liposarcoma, *EWSR1-ERG* in Ewing sarcoma, and the *EPC-PHF1*, *JAZF1-PHF1*, and *MEAF6-PHF1* fusions in endometrial stromal sarcoma [36–39]. No material was available for FISH examinations to identify on which derivative chromosome the *KAT6B-KANSL1* fusion was located. Presumably, though this is something we cannot know, the *KAT6B-KANSL1* fusion also existed in the four uterine leiomyomas with rearrangements of 10q and 17q and disruption of the *KAT6B* previously reported by Moore et al. [15]. The orientation of *KAT6B* in relation to *KANSL1* may also explain the low frequency of the t(10;17)(q22;q21). However, the frequency of *KAT6B-KANSL1* fusion might be higher as it could be a pathogenetic candidate of partially cryptic rearrangements of chromosome band 17q21 in the 34 uterine leiomyomas with cytogenetic aberrations involving the q arm of chromosome 10 (especially in the 25 such tumors with the breakpoint assigned to chromosome band 10q22) reported in the Mitelman Database of Chromosome Aberrations and Gene Fusions in Cancer (http://cgap.nci.nih.gov/Chromosomes/Mitelman, Database last updated on May 13, 2014). Moore et al. [15] showed that one of their four studied uterine leiomyomas had a cryptic chromosome 17 rearrangement which could be found by interphase FISH but not by GTG-banding.

Since the three genes *HMGA2*, *HMGA1*, and *MED12* are known to be frequently involved in the development of uterine leiomyomas, we investigated them, too, in the present tumor with a t(10;17)(q22;q21). *HMGA2* is overexpressed in leiomyomas both with and without chromosome rearrangement of 12q14–15 [40–42] suggesting a general role of this gene in leiomyoma development [41]. Similarly, overexpression of *HMGA1* has been found in uterine leiomyomas both with and without microscopically visible 6p21 aberration [43, 44]. A number of recent studies have reported mutations in exon 2 of the *MED12* gene in the majority of uterine leiomyomas (50–70%) [9–13]. Mutations of *MED12* were found in uterine leiomyomas with normal karyotype, deletions or rearrangements of the long arm of chromosome 7 as sole anomaly or 6p21~23 abnormalities leading to *HMGA1* rearrangement/overexpression, and it was concluded that the *MED12* mutations had preceded the chromosomal aberrations [45]. Mutations of *MED12* have, on the other hand, not been detected in uterine leiomyomas with 12q14~15 rearrangements resulting in overexpression of *HMGA2* [45, 46]. According to Markowski et al. [45], these data “stratify two mutually exclusive pathways of leiomyomagenesis with either rearrangements of *HMGA2* reflected by clonal chromosome abnormalities affecting 12q14~15 or by mutations affecting exon 2 of *MED12*”. Because uterine leiomyomas with *MED12* mutations expressed significantly higher levels of the gene encoding wingless-type MMTV integration site family, member 4 (*WNT4*), the authors suggested that the *MED12* mutations exert their effects by activating the canonical Wnt pathway [45]. In a recent study, *MED12* mutations were also found in 34% of leiomyoma/leiomyomatosis in pelvic/retroperitoneal sites but there was no information about the status of *HMGA2* and *HMGA1* [9].

The examined retroperitoneal leiomyoma in addition to the *KAT6B-KANSL1* fusion transcript also expressed *HMGA2* and *HMGA1* although there were no cytogenetically detectable aberrations of 12q14–15 (*HMGA2*) and 6p (*HMGA1*). No evidence of mutations in exon 2 of *MED12* was seen. The *KAT6B-KANSL1* fusion gene might, thus, represent a third pathway of leiomyomatogenesis involving the MLL1 and NSL1 protein complexes and histone acetylation but without chromosomal changes targeting *HMGA*-genes and *MED12*-mutations. Obviously, additional cases must be studied before conclusions can be drawn.

## Supporting Information

S1 Table Fusion transcripts detected using FusionMap. (XLSX)

S2 Table Fusion transcripts detected using FusionFinder. (XLSX)
